# Supplementary material for: Evolutionary Adaptations in Biliverdin Reductase B: Insights into Coenzyme Dynamics and Catalytic Efficiency
Source: Int J Mol Sci. 2024 Dec 10;25(24):13233. doi: 10.3390/ijms252413233 (PMC11675717; doi:10.3390/ijms252413233)
Supplement: Supplementary file 1 [file ijms-25-13233-s001.zip › ijms-3318252-supplementary.pdf]

Supplementary Data

Title: “Evolutionary Adaptations in Biliverdin Reductase B: Insights into Coenzyme Dynamics and Catalytic Efficiency”

Eunjeong Lee, Jasmina S. Redzic, and Elan Zohar Eisenmesser.

SUPPLEMENTARY FIGURES

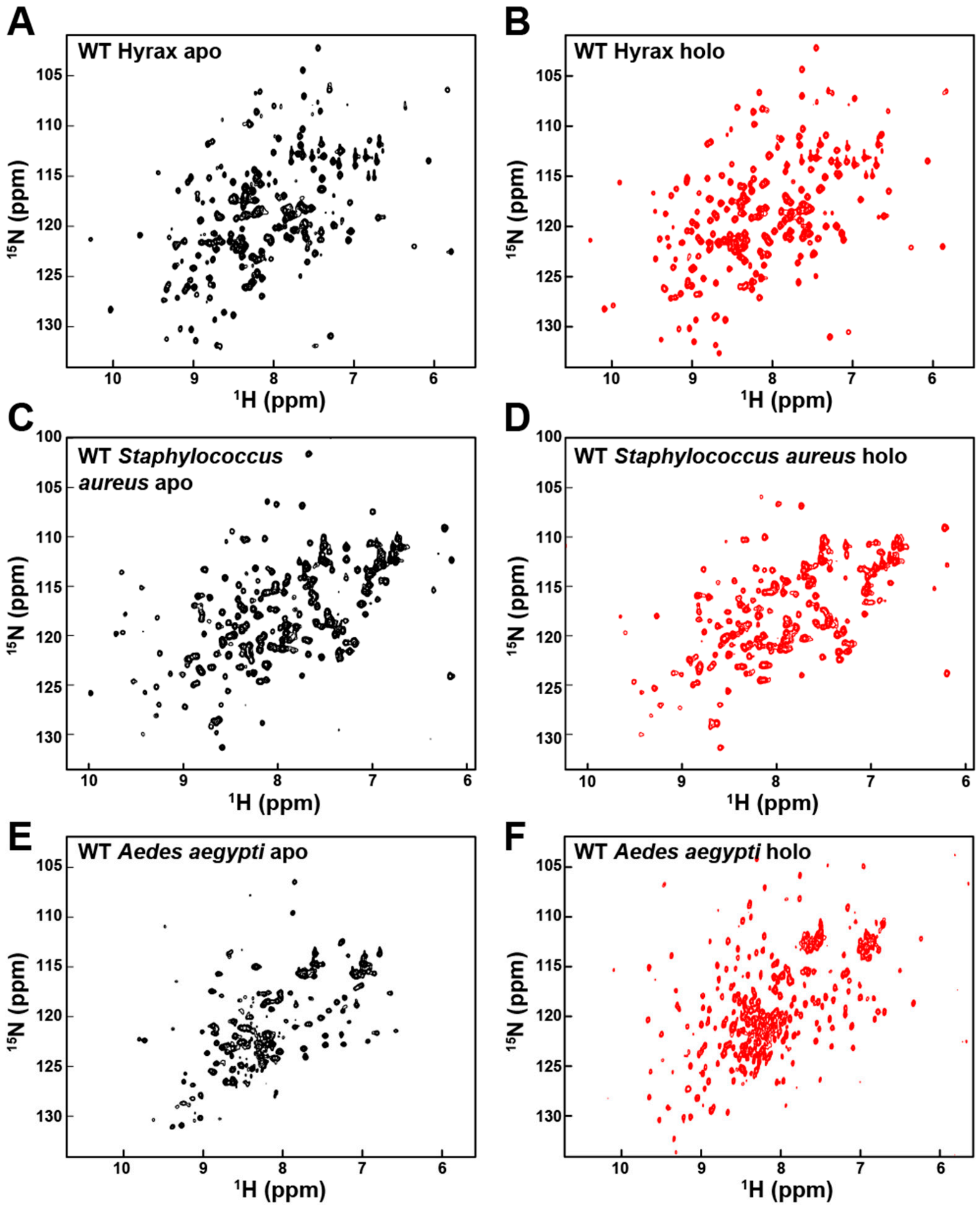

Supplementary Figure S1. Comparison of 2D ^{15}N-HSQC spectra between apo and holo states of BLVRB homologs.

A) ^{15}N-HSQC spectra of ^{15}N-labeled apo hyrax BLVRB.
B) ^{15}N-HSQC spectra of ^{15}N-labeled holo hyrax BLVRB.
C) ^{15}N-HSQC spectra of ^{15}N-labeled apo *Staphylococcus aureus* BLVRB.
D) ^{15}N-HSQC spectra of ^{15}N-labeled holo *Staphylococcus aureus* BLVRB.
E) ^{15}N-HSQC spectra of ^{15}N-labeled apo *Aedes aegypti* BLVRB.
F) ^{15}N-HSQC spectra of ^{15}N-labeled holo *Aedes aegypti* BLVRB.

All ^{15}N-HSQCs are collected at 20 °C at 900 MHz.

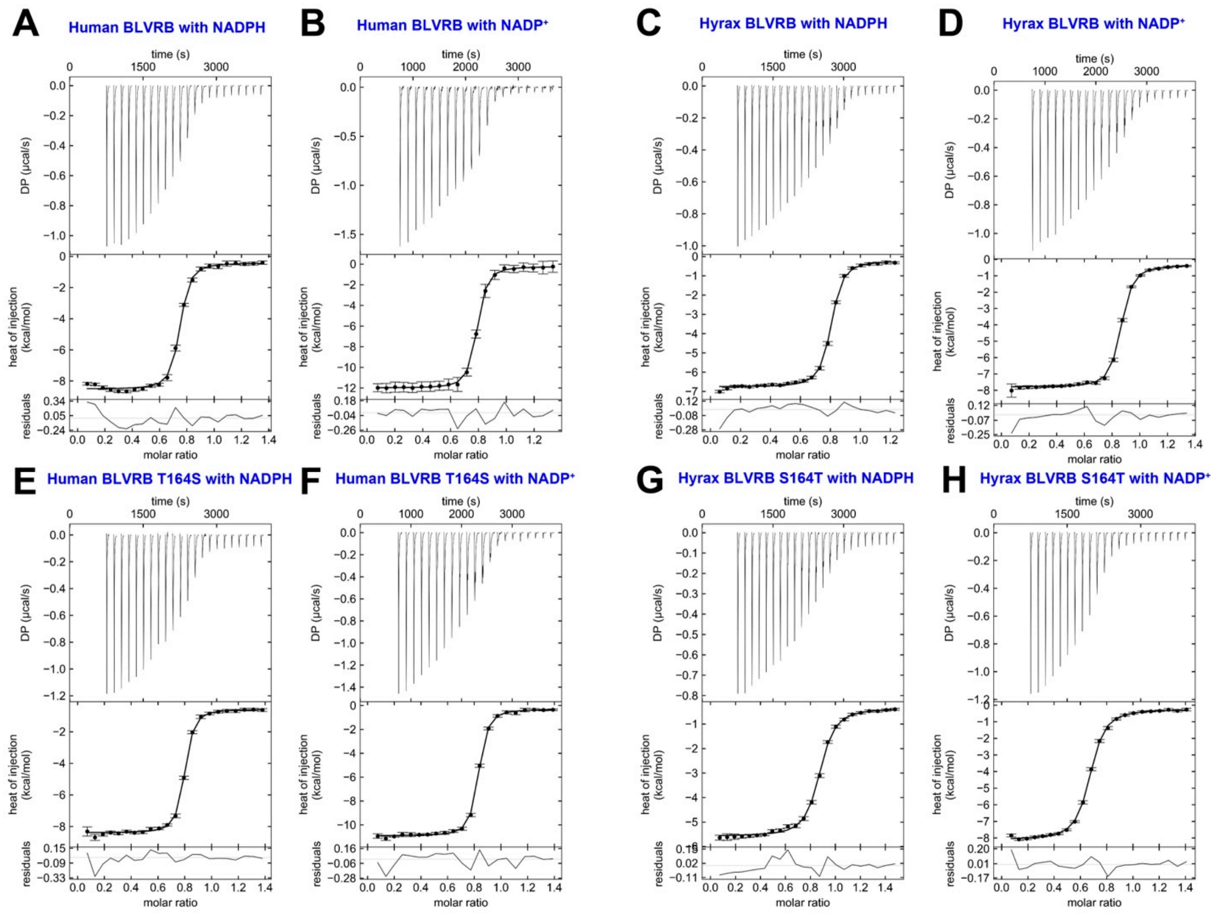

Supplementary Figure S2. Raw ITC data and fits with both oxidized and reduced coenzymes. All ITC titrations were collected at 20 °C and include the following:

A) Human BLVRB with NADPH as the titrant.
B) Human BLVRB with $NADP^+$ as the titrant.
C) Hyrax BLVRB with NADPH as the titrant.
D) Hyrax BLVRB with $NADP^+$ as the titrant.
E) Human BLVRB T164S with NADPH as the titrant.
F) Human BLVRB T164S with $NADP^+$ as the titrant.
G) Hyrax BLVRB S164T with NADPH as the titrant.
H) Hyrax BLVRB S164T with $NADP^+$ as the titrant.

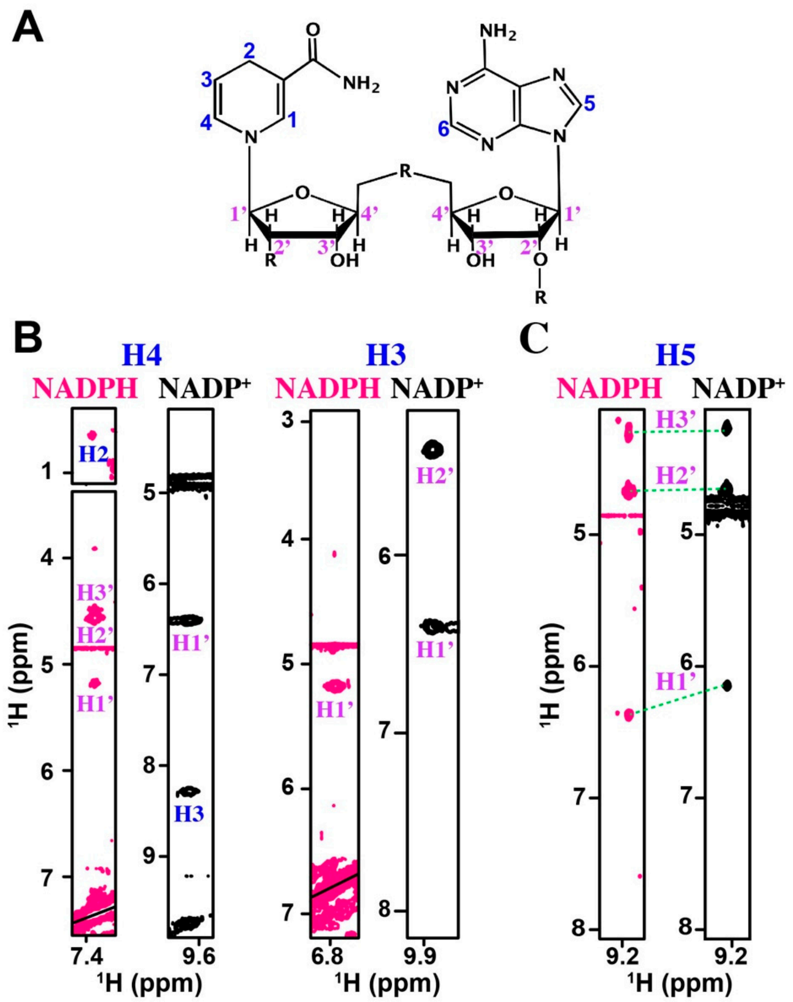

Supplementary Figure S3. 2D-filtered NOESY spectra of human BLVRB-bound NADPH and $NADP^+$.

A) Chemical structure of the NADPH coenzyme highlighting key protons on the nicotinamide, adenosine, and sugar rings.

B) Representative strip plots of the nicotinamide H4 and H3 from the 2D ^{13}C-filtered NOESY spectrum of human BLVRB with unsaturated coenzyme, comparing NADPH (magenta) and $NADP^+$ (black).

C) Adenosine H5 strip plot showing NOE correlations for NADPH and NADP+.

All spectra were collected at 20°C.

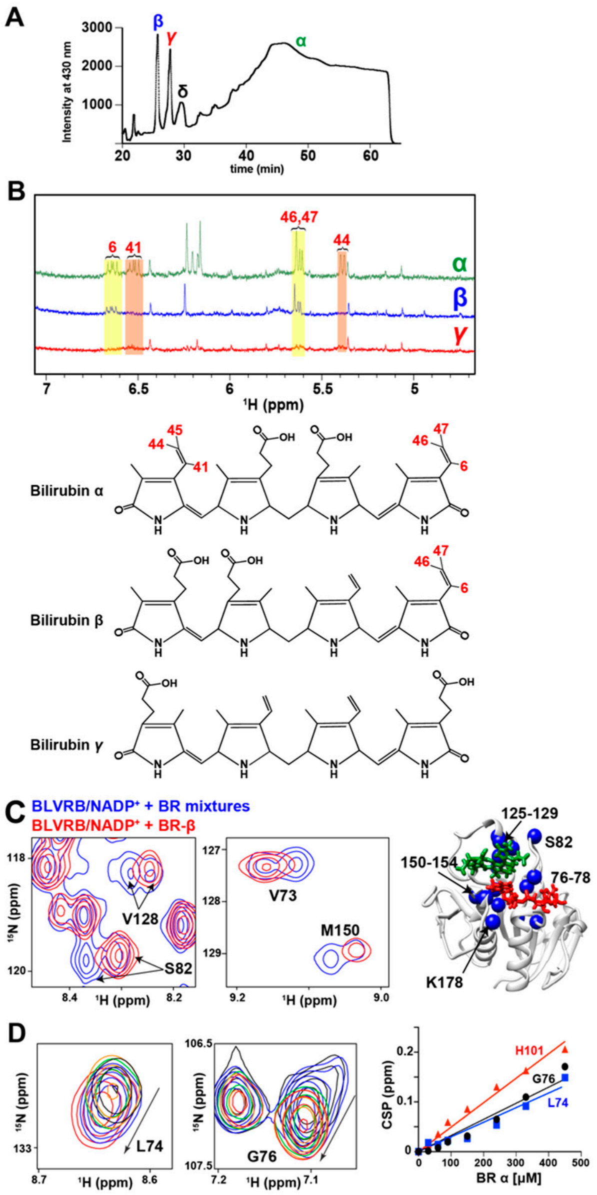

Supplementary Figure S4. Bilirubin (BR) purification and binding studies of BLVRB with BR isomers.

A) RP-HPLC chromatogram of BR mixture.

B) ^{1}H NMR spectrum of BR isomers purified by HPLC along with the structure of BR isomers: α (top), β (middle), γ (bottom). Structural differences between isomers are highlighted in the NMR spectrum with the corresponding numbering.

C) The CSPs observed in human BLVRB upon the addition of mixed BR isomers (blue) and purified BR β (red) indicate distinct chemical environments for each isomer, evidenced by the presence of two peaks. Mapping of residues that exhibit two peaks upon BR mixture binding onto the ternary structure of BLVRB (right).

D) Blown-up image of ^{15}N-HSQC spectra of ^{15}N-labeled human BLVRB upon the addition of purified BR α; corresponding binding isotherm from NMR titration is displayed on the right.

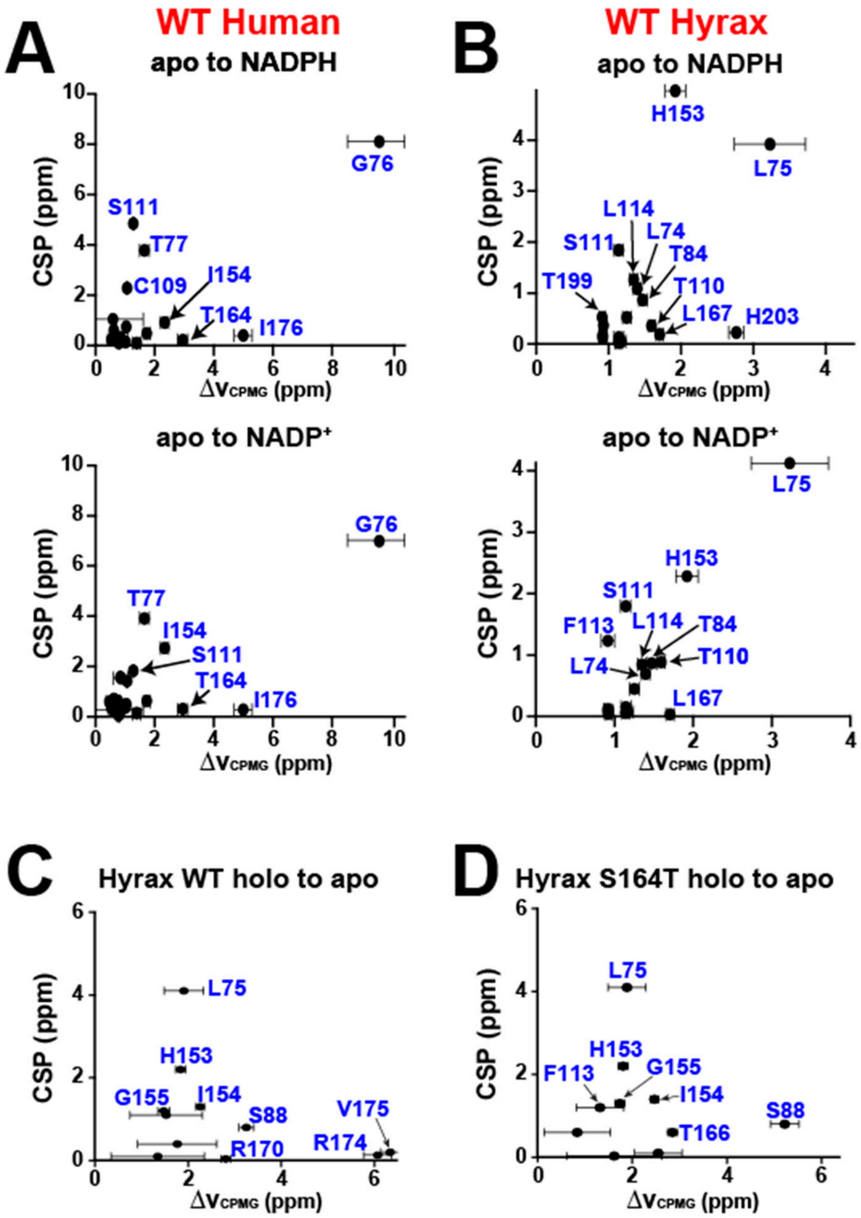

Supplementary Figure S5. Comparison between R2-CPMG-derived chemical shifts ($\Delta\nu_{CPMG}$) and experimental chemical shifts (CSPs).

A) Apo human BLVRB $\Delta\nu_{CPMG}$ measured by R2-CPMG versus CSPs between apo and NADPH-bound (top) and $NADP^+$-bound (bottom).

B) Apo hyrax BLVRB $\Delta\nu_{CPMG}$ measured by R2-CPMG versus CSPs between apo and NADPH-bound (top) and $NADP^+$-bound (bottom).

C) Holo ($NADP^+$) hyrax BLVRB $\Delta\nu_{CPMG}$ measured by R2-CPMG versus CSPs between holo and apo forms.

D) Holo ($NADP^+$) hyrax BLVRB S164T mutant $\Delta\nu_{CPMG}$ measured by R2-CPMG versus CSPs between holo and apo forms.

SUPPLEMENTARY TABLES

Table S1. Exchange rates, populations, and $\Delta\omega_N$ measured by R2-CPMG for WT apo human BLVRB and comparison to experimental CSP_N between apo and holo forms.

WT apo human	k_{ex} (Hz)	P_A (%)	$\Delta\omega_N$ (ppm, CPMG)	CSP_N (ppm, NADPH)	CSP_N (ppm, $NADP^+$)
L74	3380 ± 650	71.3 ± 20.9	0.59 ± 1.02	1.04	0.286
G76	3260 ± 1250	99.8 ± 0.8	9.49 ± 0.96	8.11	7.02
T77	1366 ± 100	96 ± 0.8	1.65 ± 0.16	3.78	3.93
D80	2130 ± 400	62.4 ± 21.3	0.54 ± 0.11	0.24	0.34
C109	2080 ± 400	50 ± 22.2	1.07 ± 0.04	2.28	1.41
S111	1420 ± 100	55.1 ± 18.8	1.28 ± 0.03	4.85	1.82
F113	10380 ± 4380	55.8 ± 22.8	0.84 ± 0.04	-	1.57
T119	2250 ± 1970	99.1 ± 21.3	0.83 ± 0.04	0.15	0.18
L125	2670 ± 500	98.8 ± 18.9	0.76 ± 0.04	-	0.64
Q126	2080 ± 250	99.2 ± 0.1	1.02 ± 0.04	0.15	0.39
A127	2900 ± 800	60.5 ± 20.3	0.62 ± 0.06	0.61	0.68
I133	1520 ± 100	91.9 ± 20.8	1.04 ± 0.04	0.75	0.47
M135	1540 ± 300	50 ± 21.6	0.66 ± 0.05	0.38	0.31
Y146	2800 ± 700	99.7 ± 0.1	0.78 ± 0.04	0.15	0.08
V147	4140 ± 900	99.7 ± 0.0	0.78 ± 0.04	0.11	0.03
I154	3030 ± 630	98.6 ± 15.9	2.34 ± 0.13	0.92	2.74
T164	8070 ± 3600	52.5 ± 21.7	2.95 ± 0.17	0.22	0.28
I176	2420 ± 500	98.7 ± 0.2	5.0 ± 0.3	0.4	0.27
K178	3000 ± 880	50 ± 21.4	1.39 ± 0.13	0.1	0.12
Q205	2220 ± 370	50 ± 19.4	1.73 ± 0.12	0.49	0.61

Table S2. Exchange rates, populations, and $\Delta\omega_N$ measured by R2-CPMG for WT apo hyrax BLVRB and comparison to experimental CSP_N between apo and holo forms.

WT apo hyrax	k_{ex} (Hz)	P_A (%)	$\Delta\omega_N$ (ppm, CPMG)	CSP_N (ppm, NADPH)	CSP_N (ppm, $NADP^+$)
L74	9560 ± 1140	99.2 ± 8.3	1.40 ± 0.05	1.09	0.69
L75	2150 ± 1680	99.8 ± 10.4	3.23 ± 0.92	3.92	4.13
T84	5150 ± 730	50 ± 20.8	1.47 ± 0.06	0.86	0.87
T110	6520 ± 3090	50 ± 20.8	1.60 ± 0.05	0.36	0.88
S111	1340 ± 400	99.7 ± 0.0	1.14 ± 0.06	1.85	1.80
F113	2520 ± 1800	99.7 ± 13.9	0.92 ± 0.08	-	1.23
L114	5180 ± 2000	99.6 ± 0.5	1.35 ± 0.05	1.27	0.85
M135	7250 ± 4100	98.4 ± 21.6	1.25 ± 0.06	0.52	0.45
Y146	2600 ± 2500	99.8 ± 8.0	1.15 ± 0.07	0.13	0.16
V147	5370 ± 1100	99.8 ± 0.0	1.17 ± 0.06	0.05	0.07
H153	1450 ± 170	98.4 ± 11.8	1.92 ± 0.14	5.57	2.29
V165	780 ± 120	99.5 ± 0.0	1.14 ± 0.05	0.03	0.06
L167	1750 ± 240	99.1 ± 0.2	1.71 ± 0.05	0.19	0.03

A199	1180.1 ± 400	99.6 ± 8.3	0.91 ± 0.04	0.53	0.12
Y200	1300 ± 100	99.6 ± 0.0	0.92 ± 0.04	0.18	0.7
C202	1170 ± 300	99.7 ± 0.1	0.93 ± 0.09	0.37	0.09
H203	1050 ± 120	98.5 ± 0.1	2.77 ± 0.10	0.23	-
N205	1177 ± 60	98.5 ± 0.1	0.92 ± 0.04	0.12	0.04

Table S3. Exchange rates, populations, and $\Delta\omega_N$ measured by R2-CPMG for WT holo hyrax BLVRB with NADP$^+$ and comparison to experimental CSP$_N$ between holo and apo forms.

WT holo hyrax	k_{ex} (Hz)	P_B (%)	$\Delta\omega_N$ (ppm, CPMG)	CSP$_N$ (ppm, apo)
L75	770 ± 320	0.25 ± 0.06	1.91 ± 0.42	4.1
S88	540 ± 140	0.45 ± 0.08	3.25 ± 0.16	0.8
F113	1370 ± 440	0.56 ± 0.51	1.53 ± 0.78	1.1
A119	1260 ± 460	0.39 ± 0.13	2.24 ± 0.59	-
Q126	1550 ± 510	0.18 ± 0.15	1.77 ± 0.85	0.4
H153	1190 ± 80	1.10 ± 0.10	1.84 ± 0.11	2.2
I154	800 ± 80	0.83 ± 0.04	2.26 ± 0.09	1.3
G155	510 ± 100	0.54 ± 0.05	1.48 ± 0.13	1.2
L167	2470 ± 370	2.20 ± 0.30	1.35 ± 1.02	0.1
R170	1070 ± 80	0.91 ± 0.03	2.81 ± 0.10	0.04
R174	2010 ± 280	1.10 ± 0.10	6.07 ± 0.31	0.13
V175	1840 ± 210	1.40 ± 0.10	6.34 ± 0.23	0.2

Table S4. Exchange rates, populations, and $\Delta\omega_N$ measured by R2-CPMG for holo hyrax BLVRB S164T mutant with NADP$^+$ and comparison to experimental CSP$_N$ between holo and apo forms.

S164T holo hyrax	k_{ex} (Hz)	P_B (%)	$\Delta\omega_N$ (ppm, CPMG)	CSP$_N$ (ppm, apo)
L75	1180 ± 290	0.27 ± 0.08	1.89 ± 0.40	4.1
S88	630 ± 350	0.25 ± 0.11	5.22 ± 0.31	0.8
F113	1360 ± 270	1.57 ± 1.24	1.32 ± 0.55	1.2
A119	4020 ± 2150	0.24 ± 0.06	6.31 ± 1.93	0.14
L125	1170 ± 70	1.30 ± 0.18	1.46 ± 0.12	-
Q126	1150 ± 250	1.04 ± 1.78	0.84 ± 0.74	0.6
H153	1125 ± 80	1.20 ± 0.11	1.81 ± 0.11	2.2
I154	1050 ± 80	1.13 ± 0.05	2.47 ± 0.09	1.4
G155	850 ± 80	0.88 ± 0.06	1.74 ± 0.10	1.3
T166	1320 ± 100	1.27 ± 0.06	2.85 ± 0.11	0.6
L167	1430 ± 470	0.87 ± 0.25	2.55 ± 0.57	0.1
Y200	2550 ± 480	0.76 ± 1.01	1.62 ± 1.09	0.03
